# New analytic bending, buckling, and free vibration solutions of rectangular nanoplates by the symplectic superposition method

**DOI:** 10.1038/s41598-021-82326-w

**Published:** 2021-02-03

**Authors:** Xinran Zheng, Mingqi Huang, Dongqi An, Chao Zhou, Rui Li

**Affiliations:** State Key Laboratory of Structural Analysis for Industrial Equipment, Department of Engineering Mechanics, and International Research Center for Computational Mechanics, Dalian University of Technology, Dalian, 116024 China

**Keywords:** Mechanical engineering, Applied mathematics

## Abstract

New analytic bending, buckling, and free vibration solutions of rectangular nanoplates with combinations of clamped and simply supported edges are obtained by an up-to-date symplectic superposition method. The problems are reformulated in the Hamiltonian system and symplectic space, where the mathematical solution framework involves the construction of symplectic eigenvalue problems and symplectic eigen expansion. The analytic symplectic solutions are derived for several elaborated fundamental subproblems, the superposition of which yields the final analytic solutions. Besides Lévy-type solutions, non-Lévy-type solutions are also obtained, which cannot be achieved by conventional analytic methods. Comprehensive numerical results can provide benchmarks for other solution methods.

## Introduction

Nanoplates play an important role in the micro- and nano-scale technology, with applications to nano sensors, resonators, storage components, micro switches, etc. Mechanical behaviors, including bending, buckling, and free vibration, are frequently encountered in response to external excitations during the use of nanoplates. Accordingly, investigations on such behaviors are crucial for understanding the mechanical properties as well as providing guidelines for structural safety designs of relevant devices. In order to avoid enormous computational efforts when carrying out discrete atomistic or molecular dynamics simulation^1–5^, some continuum theories considering scale effects, which were not incorporated in classical plate theories, have been proposed, including the couple stress elasticity theory^6^, strain gradient theory^7^, micro-morphic theory^8^, surface energy incorporated continuum theory^9^, etc. One of the well-accepted models is the non-local continuum theory by Eringen^10^, which assumes that the stress at a point is a function of the strains at all the other points in the domain. Lu et al.^11^ established the non-local elastic plate theories based on Eringen’s theory, where the basic equations for the non-local Kirchhoff and Mindlin plate theories were derived, and the bending and free vibration problems of a rectangular nanoplate with simply supported edges were solved. It was shown that, for very small-sized plates, the influences of non-local effects on the mechanical properties are considerable.

Many studies have been conducted on modeling two-dimensional plate-like structures using the nonlocal plate theories. Some notable progresses by numerical methods are briefly reviewed in the following. Pradhan and Murmu^12^ explored the small-scale effect on the buckling analysis of biaxially compressed simply supported single-layered graphene sheets (SLGS) by computing the buckling loads using the differential quadrature method (DQM), which was also employed for vibration analysis of SLGS embedded in elastic medium^13^. Malekzadeh and Shojaee^14^ extended a two-variable refined plate theory for the free vibration of nanoplates, where different types of boundary conditions (BCs) were studied by the DQM. Farajpour et al.^15,16^ used the DQM to analyze the buckling of higher-order and lower-order nonlocal strain gradient theory based orthotropic micro/nanoscale plates. Mohammadsalehi et al.^17^ investigated the vibration features of rectangular viscoelastic nanoplates with variable thickness by the DQM. Ghadiri et al.^18^ applied the generalized DQM to investigate the thermo-mechanical vibration of orthotropic cantilever and propped cantilever nanoplates. The method was also adopted by Ebrahimi et al.^19^ to analyze the thermo-mechanical vibration of rotating nonlocal nanoplates. Phadikar and Pradhan^20^ reported finite element formulations for nonlocal elastic Euler–Bernoulli beam and Kirchhoff plate, and analyzed bending, vibration, and buckling of simply supported nonlocal plates. Bahu and Patel^21^ developed an improved quadrilateral finite element for nonlinear second-order strain gradient elastic Kirchhoff plates based on the nonlocal theory. Necira^22^ developed the hierarchical finite element method for size-dependent free vibration analysis of Mindlin nano-plates with curvilinear plan-forms. Akgöz and Civalek^23^ employed the discrete singular convolution method for the free vibration and bending analysis of nano-scaled graphene sheets having sector shape. Babaei and Shahidi^24^ investigated the buckling behavior of quadrilateral SLGS under bi-axial compression by the Galerkin method, where the buckling loads of nanoplates with different geometrical parameters were obtained. Rahimi et al.^25^ studied the thermoelastic damping of in-plane vibration of the functionally graded nanoplates using the Galerkin method. Based on three-dimensional nonlocal elasticity theory, Shahrbabaki^26^ developed novel trigonometric series to be used as approximating functions in the Galerkin based approach in dealing with free vibration problems of rectangular nanoplates. Anjomshoa^27^ adopted the Ritz functions for buckling analysis of embedded orthotropic circular and elliptical micro/nano-plates under uniform in-plate compression. Chacraverty and Behera^28^ took the Rayleigh–Ritz method with algebraic polynomial displacement function to solve the vibration problem of isotropic rectangular nanoplates subjected to different BCs. Analooei et al.^29^ addressed the buckling and vibration characteristics of isotropic and orthotropic nanoplates using the spline finite strip method (FSM). Sarrami-Foroushani and Azhari^30^ examined the vibration and buckling characteristics of single and multi-layered graphene sheets by the FSM. Wang et al.^31^ presented highly accurate solutions for free vibration and eigen buckling of rectangular nanoplates with the iterative separation-of-variable (iSOV) method. Thanh et al.^32–35^ conducted the bending, buckling, and vibration analyses of microplates via the isogeometric method with couple stress theory, and further extended the method to the thermal buckling and post-buckling analyses of functionally graded micro-plates with porosities.

Although various effective numerical methods have been developed to study the mechanical behaviors of nanoplates, it is still important to explore new analytic methods because they cannot only provide benchmark theoretical solutions of permanent interests, but can also explicitly capture the relationships among different mechanical quantities, thus can serve as useful tools for validation of numerical methods, rapid parameter analyses, and efficient structural designs. However, due to the recognized mathematical difficulties in solving the complex boundary-value problems of governing higher-order partial differential equations (PDEs), the applicability of conventional analytic methods is generally restricted to some specific cases such as Navier-type and Lévy-type rectangular nanoplates, i.e., those fully simply supported or with at least two parallel edges simply supported. Some representative studies in this regard are briefly reviewed here. Aghababaei and Reddy^36^ reformulated the third-order shear deformation plate theory using the nonlocal theory, and presented analytical solutions of bending and free vibration of a simply supported rectangular nanoplate. Aksencer and Aydogdu^37^ used Navier-type solution and Lévy-type solution for vibration and buckling of simply supported nanoplates and those with two opposite edges simply supported. Sumelka^38^ proposed fractional calculus as a new formulation to study the nonlocal Kirchhoff–Love plates, taking the case of simply supported plate as an illustrative example. Based on Reddy’s nonlocal third-order shear deformation plate theory, Hosseini-Hashemi et al.^39^ obtained Lévy-type solutions for buckling and vibration problems of rectangular nanoplates. Ilkhani et al.^40^ applied the wave propagation approach to determine the natural frequencies of rectangular nanoplates with two opposite edges simply supported. Jamalpoor et al.^41^ adopted the Navier approach to solve free vibration and biaxial buckling of double-magneto-electro-elastic nanoplate-systems subjected to initial external electric and magnetic potentials. Moradi-Dasjerdi et al.^42^ applied the Navier approach at the free vibration analysis of nanocomposite sandwich plates reinforced with CNT aggregates. Arefi et al.^43^ adopted the Navier-form solutions to analyze the free vibration of a sandwich nano-plate including FG core and piezoelectric face-sheets. Cornacchia et al.^44^ obtained the Navier solutions for vibration and buckling of Kirchhoff nanoplates using second-order strain gradient theory. Besides, Yang et al.^45^ utilized the Bessel functions to settle the bending problems of circular nanoplates under concentrated and uniform loads.

In recent years, we have proposed an analytic symplectic superposition method for mechanics problems of plates and shells based on classical theories, which proved to be widely applicable to bending^46,47^, buckling^48^, and vibration^49,50^ problems. The solution procedure involves three main steps, i.e., converting an original problem into several elaborated subproblems, solving the subproblems within the Hamiltonian system by the symplectic approach, and superposition of the subproblems for the final solution. Specifically, the symplectic eigenvalue problems of a Hamiltonian matrix are introduced, followed by symplectic eigen expansion, to yield the analytic solutions of the subproblems, which are exclusive mathematical techniques in the symplectic space^51^ rather than in the traditional Euclidean space. However, since the governing equations of the nanoplate problems are much more complex than those of the classical plate problems, there has been almost no research on developing the symplectic superposition method for analytic modeling of similar issues. In the following, for the first time, the symplectic superposition method is extended to obtain the analytic bending, buckling, and free vibration solutions of rectangular nanoplates with all combinations of clamped and simply supported edges, including both Lévy-type and non-Lévy-type solutions. Comprehensive benchmark results are presented to show fast convergence and high accuracy of the present solutions by excellent agreement with those obtained by the finite element method (FEM) and other numerical methods in the open literature. The effects of the nonlocal parameter and plate dimensions on the mechanical behaviors of the nanoplates are well examined with the present analytic solutions. Some useful conclusions are drawn to reflect the small-scale effects that are not captured in classical theories.

## Governing equation for bending, buckling, and free vibration of nanoplates in the Hamiltonian system

Based on the nonlocal theory by Eringen^10^, the transformed differential constitutive equation^27^ is

1 $$\sigma_{ij}^{n} - \mu \nabla^{2} \sigma_{ij}^{n} = \sigma_{ij} = S_{ijkl} \varepsilon_{kl}$$

where $\sigma_{ij}$, $\sigma_{ij}^{n}$, $S_{ijkl}$, and $\varepsilon_{kl}$ denote the components of local stress tensor, nonlocal stress tensor, fourth order stiffness tensor and strain tensor, respectively, $\mu = \left( {e_{0} l} \right)^{2}$ is the nonlocal parameter depending on the internal characteristic length, *l*, and an experimentally defined material constant, $e_{0}$. For isotropic thin nanoplates, we have

2 $$\left\{ {\begin{array}{*{20}c} {\sigma_{x}^{n} } \\ {\sigma_{y}^{n} } \\ {\sigma_{xy}^{n} } \\ \end{array} } \right\} - \mu \nabla^{2} \left\{ {\begin{array}{*{20}c} {\sigma_{x}^{n} } \\ {\sigma_{y}^{n} } \\ {\sigma_{xy}^{n} } \\ \end{array} } \right\} = \left\{ {\begin{array}{*{20}c} {\sigma_{x}^{{}} } \\ {\sigma_{y}^{{}} } \\ {\sigma_{xy}^{{}} } \\ \end{array} } \right\} = \left[ {\begin{array}{*{20}c} {\frac{E}{{1 - \nu^{2} }}} & {\frac{\nu E}{{1 - \nu^{2} }}} & 0 \\ {\frac{\nu E}{{1 - \nu^{2} }}} & {\frac{E}{{1 - \nu^{2} }}} & 0 \\ 0 & 0 & {\frac{E}{{2\left( {1 + \nu } \right)}}} \\ \end{array} } \right]\left\{ {\begin{array}{*{20}c} {\varepsilon_{x} } \\ {\varepsilon_{y} } \\ {\varepsilon_{xy} } \\ \end{array} } \right\}$$

where *E* and $\nu$ are Young’s modulus and Poisson’s ratio, respectively. The stress resultants are defined as

3 $$\bf {\mathbf{\rm M}}^{n} = \left\{ {\begin{array}{*{20}c} {M_{x}^{n} } \\ {M_{y}^{n} } \\ {M_{xy}^{n} } \\ \end{array} } \right\} = \int_{{{{ - h} \mathord{\left/ {\vphantom {{ - h} 2}} \right. \kern-\nulldelimiterspace} 2}}}^{{{h \mathord{\left/ {\vphantom {h 2}} \right. \kern-\nulldelimiterspace} 2}}} {z\left\{ {\begin{array}{*{20}c} {\sigma_{x}^{n} } \\ {\sigma_{y}^{n} } \\ {\sigma_{xy}^{n} } \\ \end{array} } \right\}{\text{d}}z}$$

where *h* is the thickness of the nanoplate. The moment component resultants are thus

4 $$\left\{ {\begin{array}{*{20}c} {M_{x}^{n} } \\ {M_{y}^{n} } \\ {M_{xy}^{n} } \\ \end{array} } \right\} - \mu \nabla^{2} \left\{ {\begin{array}{*{20}c} {M_{x}^{n} } \\ {M_{y}^{n} } \\ {M_{xy}^{n} } \\ \end{array} } \right\} = - \left[ {\begin{array}{*{20}c} D & {\nu D} & 0 \\ {\nu D} & D & 0 \\ 0 & 0 & {\frac{D}{{2\left( {1 + \nu } \right)}}} \\ \end{array} } \right]\left\{ {\begin{array}{*{20}c} {\varepsilon_{x} } \\ {\varepsilon_{y} } \\ {\varepsilon_{xy} } \\ \end{array} } \right\}$$

where $D = {{Eh^{3} } \mathord{\left/ {\vphantom {{Eh^{3} } {\left[ {12\left( {1 - \nu^{2} } \right)} \right]}}} \right. \kern-\nulldelimiterspace} {\left[ {12\left( {1 - \nu^{2} } \right)} \right]}}$ is the bending stiffness.

**Figure 1:**
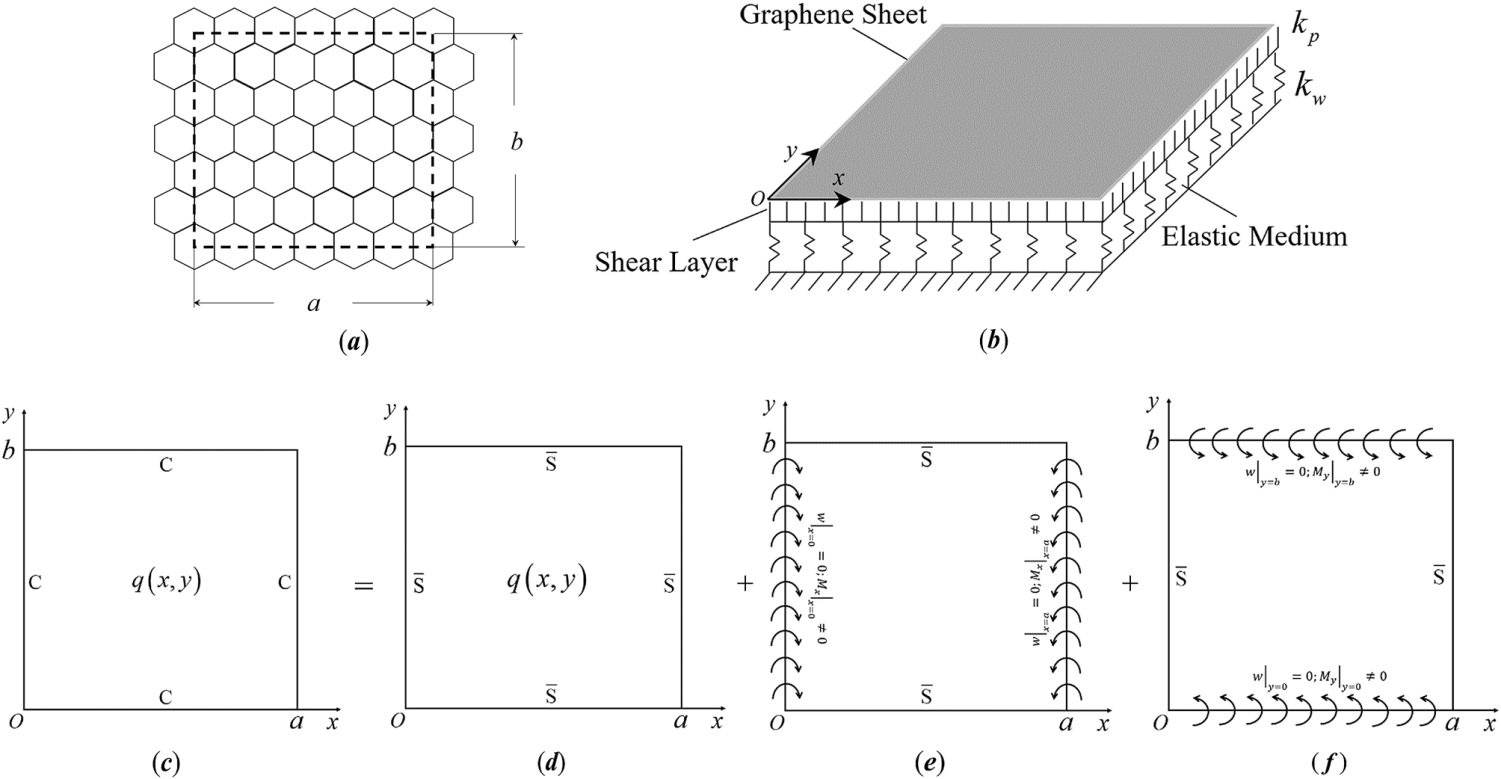
(**a**) Top view and (**b**) tilted side view of a rectangular nanoplate resting on a two-parameter elastic foundation. (**c–f**) Symplectic superposition for a fully clamped rectangular nanoplate, where the original problem (**c**) is equivalent to the superposition of the three subproblems (**d**–**f**).

The nonlocal theory-based quadratic functional for a nanoplate within the domain $\Omega$ resting on a two-parameter elastic foundation as shown in Fig. 1a,b is written as^27,28,31^

5 $$\Pi_{total}^{{}} = \iint_{\Omega } {\left[ {U\left( {x,y} \right) - T\left( {x,y} \right) + V\left( {x,y} \right) - Q\left( {x,y} \right)} \right]}{\text{d}}x{\text{d}}y$$

in which

6 $$\begin{gathered} U\left( {x,y} \right) = \frac{1}{2}\left\{ {D\left[ {\left( {\frac{{\partial^{2} w}}{{\partial x^{2} }}} \right)^{2} + 2\nu \left( {\frac{{\partial^{2} w}}{{\partial x^{2} }}\frac{{\partial^{2} w}}{{\partial y^{2} }}} \right) + \left( {\frac{{\partial^{2} w}}{{\partial y^{2} }}} \right)^{2} + 2\left( {1 - \nu } \right)\left( {\frac{{\partial^{2} w}}{\partial x\partial y}} \right)^{2} } \right]} \right. \\ { + }\left. {k_{w} \left\{ {w^{2} + \mu \left[ {\left( {\frac{\partial w}{{\partial x}}} \right)^{2} + \left( {\frac{\partial w}{{\partial y}}} \right)^{2} } \right]} \right\} + k_{p} \left\{ {\left( {\frac{\partial w}{{\partial x}}} \right)^{2} + \left( {\frac{\partial w}{{\partial y}}} \right)^{2} + \mu \left[ {\left( {\frac{{\partial^{2} w}}{{\partial x^{2} }}} \right)^{2} + 2\left( {\frac{{\partial^{2} w}}{\partial x\partial y}} \right)^{2} + \left( {\frac{{\partial^{2} w}}{{\partial y^{2} }}} \right)^{2} } \right]} \right\}} \right\} \\ \end{gathered}$$

7 $$T\left( {x,y} \right) = \frac{1}{2}m_{0} \omega^{2} \left\{ {w^{2} + \mu \left[ {\left( {\frac{\partial w}{{\partial x}}} \right)^{2} + \left( {\frac{\partial w}{{\partial y}}} \right)^{2} } \right] + \frac{{m_{2} }}{{m_{0} }}\left\{ {\left( {\frac{\partial w}{{\partial x}}} \right)^{2} + \left( {\frac{\partial w}{{\partial y}}} \right)^{2} + \mu \left[ {\left( {\frac{{\partial^{2} w}}{{\partial x^{2} }}} \right)^{2} + 2\left( {\frac{{\partial^{2} w}}{\partial x\partial y}} \right)^{2} + \left( {\frac{{\partial^{2} w}}{{\partial y^{2} }}} \right)^{2} } \right]} \right\}} \right\}$$

8 $$V\left( {x,y} \right) = \frac{1}{2}\left\{ {\mu \left\{ {N_{y} \left[ {\left( {\frac{{\partial^{2} w}}{{\partial y^{2} }}} \right)^{2} + \left( {\frac{{\partial^{2} w}}{\partial x\partial y}} \right)^{2} } \right] + N_{x} \left[ {\left( {\frac{{\partial^{2} w}}{{\partial x^{2} }}} \right)^{2} + \left( {\frac{{\partial^{2} w}}{\partial x\partial y}} \right)^{2} } \right]} \right\} + N_{y} \left( {\frac{\partial w}{{\partial y}}} \right)^{2} + N_{x} \left( {\frac{\partial w}{{\partial x}}} \right)^{2} } \right\}$$

9 $$Q\left( {x,y} \right) = q\left( {x,y} \right)\left[ {w - \mu \left( {\frac{{\partial^{2} w}}{{\partial x^{2} }} + \frac{{\partial^{2} w}}{{\partial y^{2} }}} \right)} \right]$$

Here, *x* and *y* are the coordinate variables; *w* is the transverse deflection of the nanoplate; $k_{w}$ and $k_{p}$ are Winkler and Pasternak foundation coefficients, respectively; $m_{0} = \int_{ - h/2}^{h/2} \rho {\text{d}}z$, and $m_{2} = \int_{ - h/2}^{h/2} {\rho z^{2} } {\text{d}}z$, with $\rho$ being the mass density of the nanoplate; $N_{x}$ and $N_{y}$ are the membrane forces along the *x* and *y* directions, respectively; $q\left( {x,y} \right)$ is the transverse external load. Putting $\mu = 0$, the quadratic functional for the classical thin plate model is obtained.

The variation of the governing equation (5) about *w* yields

10 $$- \overline{R}_{x} \frac{{\partial^{4} w}}{{\partial x^{4} }} - \overline{R}_{y} \frac{{\partial^{4} w}}{{\partial y^{4} }} - \overline{R}_{xy} \frac{{\partial^{4} w}}{{\partial x^{2} \partial y^{2} }} + \tilde{R}_{x} \frac{{\partial^{2} w}}{{\partial x^{2} }} + \tilde{R}_{y} \frac{{\partial^{2} w}}{{\partial y^{2} }} + \left( {\overline{m}_{0} - k_{w} } \right)w + \left( {1 - \mu \nabla^{2} } \right)q = 0$$

where $$\overline{R}_{x} = D + \mu \left( {k_{p} + N_{x} - \overline{m}_{2} } \right)$$, $$\overline{R}_{y} = D + \mu \left( {k_{p} + N_{y} - \overline{m}_{2} } \right)$$, $$\overline{R}_{xy} = \overline{R}_{x} + \overline{R}_{y}$$, $$\tilde{R}_{x} = k_{p} + N_{x} - \overline{m}_{2} + \mu \left( {k_{w} - \overline{m}_{0} } \right)$$, $$\tilde{R}_{y} = k_{p} + N_{y} - \overline{m}_{2} + \mu \left( {k_{w} - \overline{m}_{0} } \right)$$, $$\overline{m}_{0} = \omega^{2} m_{0}$$, and $$\overline{m}_{2} = \omega^{2} m_{2}$$.

Define the generalized displacement vector

11 $${\mathbf{q}} = \left\{ {w,\theta_{x} } \right\}^{{\text{T}}}$$

where

12 $$\theta_{x} = - \frac{\partial w}{{\partial x}}$$

The corresponding generalized force vector is

13 $${\mathbf{p}} = \frac{{\partial \Pi_{total}^{{}} }}{{\partial {\dot{\mathbf{q}}}}} = \left\{ {\begin{array}{*{20}c} {V_{x}^{n} } \\ {M_{x}^{n} } \\ \end{array} } \right\}$$

where $$\left( {^{\cdot}} \right) ={{\partial \left( {} \right)}/ {\partial x}}$$, and

14 $$V_{x}^{n} = - \overline{R}_{x} \frac{{\partial w^{3} }}{{\partial x^{3} }} - \left( {\overline{R}_{xy} - D\nu } \right)\frac{{\partial w^{3} }}{{\partial x\partial y^{2} }}{ + }\tilde{R}_{x} \frac{\partial w}{{\partial x}} - \mu \frac{\partial q}{{\partial x}}$$

15 $$M_{x}^{n} = - \overline{R}_{x} \frac{{\partial w^{2} }}{{\partial x^{2} }} - D\nu \frac{{\partial w^{2} }}{{\partial y^{2} }} - \mu q$$

are respectively the nonlocal equivalent shear force and nonlocal bending moment in the cross sections perpendicular to the *x* axis. By coordinate exchange, we have

16 $$V_{y}^{n} = - \overline{R}_{y} \frac{{\partial w^{3} }}{{\partial y^{3} }} - \left( {\overline{R}_{xy} - D\nu } \right)\frac{{\partial w^{3} }}{{\partial x^{2} \partial y}}{ + }\tilde{R}_{y} \frac{\partial w}{{\partial y}} - \mu \frac{\partial q}{{\partial y}}$$

17 $$M_{y}^{n} = - \overline{R}_{y} \frac{{\partial w^{2} }}{{\partial y^{2} }} - D\nu \frac{{\partial w^{2} }}{{\partial x^{2} }} - \mu q$$

A new set of quantities excluding the external load are introduced as

18 $$\begin{aligned} & V_{x} = - \overline{R}_{x} \frac{{\partial w^{3} }}{{\partial x^{3} }} - \left( {\overline{R}_{xy} - D\nu } \right)\frac{{\partial w^{3} }}{{\partial x\partial y^{2} }}{ + }\tilde{R}_{x} \frac{\partial w}{{\partial x}} \\ & M_{x} = - \overline{R}_{x} \frac{{\partial w^{2} }}{{\partial x^{2} }} - D\nu \frac{{\partial w^{2} }}{{\partial y^{2} }} \\ & V_{y} = - \overline{R}_{y} \frac{{\partial w^{3} }}{{\partial y^{3} }} - \left( {\overline{R}_{xy} - D\nu } \right)\frac{{\partial w^{3} }}{{\partial x^{2} \partial y}}{ + }\tilde{R}_{y} \frac{\partial w}{{\partial y}} \\ & M_{y} = - \overline{R}_{y} \frac{{\partial w^{2} }}{{\partial y^{2} }} - D\nu \frac{{\partial w^{2} }}{{\partial x^{2} }} \\ \end{aligned}$$

Form Eq. (12),

19 $$\frac{\partial w}{{\partial x}} = - \theta_{x}$$

Form Eq. (19) and the second equation of Eq. (18),

20 $$\frac{{\partial \theta_{x} }}{\partial x} = \frac{{M_{x} }}{{\overline{R}_{x} }} + \frac{D\nu }{{\overline{R}_{x} }}\frac{{\partial^{2} w}}{{\partial y^{2} }}$$

From Eq. (19) and the first two equations of Eq. (18),

21 $$\frac{{\partial M_{x} }}{\partial x} = V_{x} - \left( {\overline{R}_{xy} - 2D\nu } \right)\frac{{\partial^{2} \theta_{x} }}{{\partial y^{2} }} + \tilde{R}_{x} \theta_{x}$$

From Eq. (10) and the first two equations of Eq. (18),

22 $$\frac{{\partial V_{x} }}{\partial x} = \left[ {\overline{R}_{y} - \frac{{\left( {D\nu } \right)^{2} }}{{\overline{R}_{x} }}} \right]\frac{{\partial^{4} w}}{{\partial y^{4} }} - \tilde{R}_{y} \frac{{\partial^{2} w}}{{\partial y^{2} }} + \left( {k_{w} - \overline{m}_{0} } \right)w - \frac{D\nu }{{\overline{R}_{x} }}\frac{{\partial^{2} M_{x} }}{{\partial y^{2} }} - \left( {1 - \mu \nabla^{2} } \right)q$$

Equations (19–22) are written in matrix form as

23 $$\frac{{\partial {\mathbf{Z}}}}{\partial x}{\mathbf{ = HZ}} + {\mathbf{f}}$$

where ${\mathbf{Z}} = \left[ {w,\theta_{x} ,V_{x} ,M_{x} } \right]^{{\text{T}}}$ is referred to as the state vector; $${\mathbf{H = }}\left[ {\begin{array}{*{20}c} {\mathbf{F}} & {\mathbf{G}} \\ {\mathbf{Q}} & { - {\mathbf{F}}^{{\text{T}}} } \\ \end{array} } \right]$$, with $${\mathbf{Q}} = \left[ {\begin{array}{*{20}c} {\left[ {\overline{R}_{y} - \frac{{\left( {D\nu } \right)^{2} }}{{\overline{R}_{x} }}} \right]\frac{{\partial^{4} }}{{\partial y^{4} }} - \tilde{R}_{y} \frac{{\partial^{2} }}{{\partial y^{2} }} + \left( {k_{w} - \overline{m}_{0} } \right)} & 0 \\ 0 & { - \left( {\overline{R}_{xy} - 2D\nu } \right)\frac{{\partial^{2} }}{{\partial y^{2} }} + \tilde{R}_{x} } \\ \end{array} } \right]$$, $${\mathbf{F}} = \left[ {\begin{array}{*{20}c} 0 & { - 1} \\ {\frac{D\nu }{{\overline{R}_{x} }}\frac{{\partial^{2} }}{{\partial y^{2} }}} & 0 \\ \end{array} } \right]$$, and $${\mathbf{G}} = \left[ {\begin{array}{*{20}c} 0 & 0 \\ 0 & {\frac{1}{{\overline{R}_{x} }}} \\ \end{array} } \right]$$; ${\mathbf{f}} = \left[ {0,0, - \left( {1 - \mu \nabla^{2} } \right)q,0} \right]^{{\text{T}}}$ is the transverse external force vector that only exists in a bending problem.** H** is a Hamiltonian operator matrix satisfying ${\mathbf{H}}^{{\text{T}}} = {\mathbf{JHJ}}$, where $${\mathbf{J}} = \left[ {\begin{array}{*{20}c} {\mathbf{0}} & {{\mathbf{I}}_{2} } \\ {{\mathbf{I}}_{2} } & {\mathbf{0}} \\ \end{array} } \right]$$ is the unit symplectic matrix with 2 × 2 unit matrix **I**_2_; accordingly, Eq. (23) gives the governing equation for bending, buckling, and free vibration of nanoplates in the Hamiltonian system.

## Fundamental analytic solutions in the symplectic space

In applying the symplectic superposition method, an original problem is converted into superposition of several elaborated subproblems that are solved in the symplectic space, whose solutions are referred to as the fundamental analytic solutions in this study.

Taking bending of a fully clamped nanoplate as an example, Fig. 1c–f schematically shows the symplectic superposition of the problem, where the nanoplate (Fig. 1c) has length *a* and width *b*, with the axes *ox* and *oy* along the plate edges. Corresponding to the bending moments excluding the external load, as expressed in Eq. (18), we denote the BCs $w = 0$, $M_{x} = 0$ at $x = 0,\;a$ and $w = 0$, $M_{y} = 0$ at $y = 0,\;b$ by “${\overline{\text{S}}}$”. In comparison, the actual simply supported conditions of a nanoplate, denoted by “S”, imply $w = 0$, $M_{x}^{n} = 0$ at $x = 0,\;a$ and $w = 0$, $M_{y}^{n} = 0$ at $y = 0,\;b$.

The first subproblem (Fig. 1d) is for a transversely loaded nanoplate with all edges ${\overline{\text{S}}}$-supported. For the second subproblem (Fig. 1e), the same ${\overline{\text{S}}}$-supported nanoplate is driven by a pair of nonzero $\left. {M_{x} } \right|_{x = 0}$ and $\left. {M_{x} } \right|_{x = a}$ that are expanded as $\sum\nolimits_{n = 1,2,3, \ldots }^{\infty } {E_{n} } \sin \left( {\beta_{n} y} \right)$ and $\sum\nolimits_{n = 1,2,3, \ldots }^{\infty } {F_{n} } \sin \left( {\beta_{n} y} \right)$, respectively. For the third subproblem (Fig. 1f), the same ${\overline{\text{S}}}$-supported nanoplate is driven by a pair of nonzero $\left. {M_{y} } \right|_{y = 0}$ and $\left. {M_{y} } \right|_{y = b}$ that are expanded as $\sum\nolimits_{n = 1,2,3, \ldots }^{\infty } {G_{n} } \sin \left( {\alpha_{n} x} \right)$ and $\sum\nolimits_{n = 1,2,3, \ldots }^{\infty } {H_{n} } \sin \left( {\alpha_{n} y} \right)$, respectively. Here, $\alpha_{n} = {{n\pi } \mathord{\left/ {\vphantom {{n\pi } a}} \right. \kern-\nulldelimiterspace} a}$, $\beta_{n} = {{n\pi } \mathord{\left/ {\vphantom {{n\pi } b}} \right. \kern-\nulldelimiterspace} b}$; $E_{n}$, $F_{n}$, $G_{n}$, and $H_{n}$ are the series expansion coefficients, which will be determined later. The BCs are thus

24 $$\begin{aligned} \left. w \right|_{x = 0,a} & = 0,\left. {M_{x} } \right|_{x = 0,a} = 0 \\ \left. w \right|_{y = 0,b} & = 0,\left. {M_{y} } \right|_{y = 0,b} = 0 \\ \end{aligned}$$

for the first subproblem,

25 $$\begin{aligned} \left. {M_{x} } \right|_{x = 0} & = \sum\nolimits_{n = 1,2,3, \ldots }^{\infty } {E_{n} } \sin \left( {\beta_{n} y} \right),\left. {M_{x} } \right|_{x = a} = \sum\nolimits_{n = 1,2,3, \ldots }^{\infty } {F_{n} } \sin \left( {\beta_{n} y} \right) \\ \left. w \right|_{x = 0,a} & = 0,\left. w \right|_{y = 0,b} = 0,\left. {M_{y} } \right|_{y = 0,b} = 0 \\ \end{aligned}$$

for the second subproblem, and

26 $$\begin{aligned} \left. w \right|_{x = 0,a} & = 0,\left. {M_{x} } \right|_{x = 0,a} = 0,\left. w \right|_{y = 0,b} = 0 \\ \left. {M_{y} } \right|_{y = 0} & = \sum\nolimits_{n = 1,2,3, \ldots }^{\infty } {G_{n} } \sin \left( {\alpha_{n} x} \right),\left. {M_{y} } \right|_{y = b} = \sum\nolimits_{n = 1,2,3, \ldots }^{\infty } {H_{n} } \sin \left( {\alpha_{n} x} \right) \\ \end{aligned}$$

for the third subproblem.

All the three subproblems come down to a general problem for a nanoplate with a pair of opposite edges ${\overline{\text{S}}}$-supported. Taking the nanoplate ${\overline{\text{S}}}$-supported at *y* = 0 and *y* = *b* as an example, the homogeneous equation of Eq. (23) is

27 $$\frac{{\partial {\mathbf{Z}}}}{\partial x}{\mathbf{ = HZ}}$$

The validity of variable separation in the symplectic space^52^ gives

28 $${\mathbf{Z}} = X\left( x \right){\mathbf{Y}}\left( y \right)$$

where $X\left( x \right)$ is a function of *x*, and

29 $${\mathbf{Y}}\left( y \right) = \left[ {w\left( y \right),\theta_{x} \left( y \right),V_{x} \left( y \right),M_{x} \left( y \right)} \right]^{{\text{T}}}$$

is a vector with argument *y*. Substituting Eq. (28) into Eq. (27) yields

30 $$\frac{{{\text{d}}X\left( x \right)}}{{{\text{d}}x}} = \xi X\left( x \right)$$

and

31 $${\mathbf{HY}}\left( y \right) = \xi {\mathbf{Y}}\left( y \right)$$

with the eigenvalue $\xi$ and the eigenvalue ${\mathbf{Y}}\left( y \right)$. With the BCs at *y* = 0 and *y* = *b*, we obtain the eigenvalues and eigenvectors:

32 $$\begin{aligned} \xi_{n1} & = - \xi_{n2} = \sqrt {\frac{{\tilde{R}_{x} + \overline{R}_{xy} \beta_{n}^{2} - \sqrt {\left( {\overline{R}_{xy} \beta_{n}^{2} + \tilde{R}_{x} } \right)^{2} - 4\overline{R}_{x} \left[ {\beta_{n}^{2} \left( {\tilde{R}_{y} + \beta_{n}^{2} \overline{R}_{y} } \right) + k_{w} - \overline{m}_{0} } \right]} }}{{2\overline{R}_{x} }}} \\ \xi_{n3} & = - \xi_{n4} = \sqrt {\frac{{\tilde{R}_{x} + \overline{R}_{xy} \beta_{n}^{2} + \sqrt {\left( {\overline{R}_{xy} \beta_{n}^{2} + \tilde{R}_{x} } \right)^{2} - 4\overline{R}_{x} \left[ {\beta_{n}^{2} \left( {\tilde{R}_{y} + \beta_{n}^{2} \overline{R}_{y} } \right) + k_{w} - \overline{m}_{0} } \right]} }}{{2\overline{R}_{x} }}} \\ \end{aligned}$$

and

33 $${\mathbf{Y}}_{ni} \left( y \right) = \sin \left( {\beta_{n} y} \right)\left[ {1, - \xi_{ni} ,\frac{{k_{w} - \overline{m}_{0} + \beta_{n}^{2} \left[ {\beta_{n}^{2} \overline{R}_{y} + \left( {\tilde{R}_{y} - D\nu \xi_{ni}^{2} } \right)} \right]}}{{\xi_{ni} }},D\nu \beta_{n}^{2} - \overline{R}_{x} \xi_{ni}^{2} } \right]^{{\text{T}}}$$

for $n =$ 1, 2, 3,$\ldots$ and $i =$ 1, 2, 3, and 4. Accordingly, we have

34 $${\mathbf{H\overline{Y}}}\left( y \right) = {\overline{\mathbf{Y}}}\left( y \right){\mathbf{M}}$$

where ${\mathbf{M}} = {\text{diag}}\left[ { \ldots ,\xi_{n1} ,\xi_{n2} ,\xi_{n3} ,\xi_{n4} , \ldots } \right]$, and ${\overline{\mathbf{Y}}}\left( y \right) = \left[ { \ldots ,{\mathbf{Y}}_{n1} \left( y \right),{\mathbf{Y}}_{n2} \left( y \right),{\mathbf{Y}}_{n3} \left( y \right),{\mathbf{Y}}_{n4} \left( y \right), \ldots } \right]$. Substituting Eqs. (28) and (34) into the Eq. (23), we have

35 $$\frac{{{\text{d}}{\mathbf{X}}\left( x \right)}}{{{\text{d}}x}} = {\mathbf{MX}}\left( x \right) + {\mathbf{G}}$$

where ${\mathbf{X}}\left( x \right) = \left[ { \ldots ,X_{n1} \left( x \right),X_{n2} \left( x \right),X_{n3} \left( x \right),X_{n4} \left( x \right), \ldots } \right]^{\rm T}$, and ${\mathbf{G}} = \left[ { \ldots ,g_{n1} ,g_{n2} ,g_{n3} ,g_{n4} , \ldots } \right]^{\rm T}$ is the column matrix of the expansion coefficients of ${\mathbf{f}}$, satisfying ${\mathbf{f}} = {\overline{\mathbf{Y}}}\left( y \right){\mathbf{G}}$. Utilizing the eigenvectors’ conjugacy and orthogonality, **G** is determined by $\int_{0}^{b} {{\overline{\mathbf{Y}}}} \left( y \right)^{\rm T} {\mathbf{J\overline{Y}}}\left( y \right){\mathbf{G}}{\text{d}}y = \int_{0}^{b} {{\overline{\mathbf{Y}}}} \left( y \right)^{\rm T} {\mathbf{Jf}}{\text{d}}y$.

For the uniform load with intensity $q_{u}$,

36 $$g_{ni}^{u} = - \frac{{q_{u} \xi_{ni} \left[ {1 - \cos \left( {n\pi } \right)} \right]}}{{n\pi \left[ {k_{w} - \overline{m}_{0} - \xi_{ni}^{4} \overline{R}_{x} + \beta_{n}^{2} \left( {\beta_{n}^{2} \overline{R}_{y} + \tilde{R}_{y} } \right)} \right]}}$$

for $n =$ 1, 2, 3,$\ldots$ ($i =$ 1, 2, 3, and 4), where the script “*u*” corresponds to uniform load. For the concentrated load with intensity $q_{c}$ at $\left( {x_{0} ,y_{0} } \right)$,

37 $$g_{ni}^{c} = - \frac{{q_{c} \xi_{ni} \sin \left( {y_{0} \beta_{n} } \right)}}{{b\left[ {k_{w} - \overline{m}_{0} - \xi_{ni}^{4} \overline{R}_{x} + \beta_{n}^{2} \left( {\beta_{n}^{2} \overline{R}_{y} + \tilde{R}_{y} } \right)} \right]}}\delta \left( {x - x_{0} } \right)$$

where the script “*c*” corresponds to concentrated load. From Eq. (35), we then obtain

38 $$X_{ni}^{u} \left( x \right) = c_{ni} e^{{\xi_{ni} x}} + \frac{{q_{u} \left[ {1 - \cos \left( {n\pi } \right)} \right]}}{{n\pi \left[ {k_{w} - \overline{m}_{0} - \xi_{ni}^{4} \overline{R}_{x} + \beta_{n}^{2} \left( {\beta_{n}^{2} \overline{R}_{y} + \tilde{R}_{y} } \right)} \right]}}$$

and

39 $$X_{ni}^{c} \left( x \right) = c_{ni} e^{{\xi_{ni} x}} - \frac{{q_{c} \xi_{ni} H\left( {x - x_{0} } \right)\sin \left( {y_{0} \beta_{n} } \right)e^{{\xi_{ni} \left( {x - x_{0} } \right)}} }}{{b\left[ {k_{w} - \overline{m}_{0} - \xi_{ni}^{4} \overline{R}_{x} + \beta_{n}^{2} \left( {\beta_{n}^{2} \overline{R}_{y} + \tilde{R}_{y} } \right)} \right]}}$$

for the cases with uniform and concentrated loads, respectively. Here, $H\left( {x - x_{0} } \right)$ is the Heaviside theta function, $c_{ni}$ are the constants to be determined by imposing the remaining BCs at $x = 0$ and $x = a$. The solution of the Eq. (23) is thus expressed by

40 $${\mathbf{Z}} = \sum\limits_{n = 1,2,3,...}^{\infty } {\sum\limits_{i = 1}^{4} {X_{ni} {\mathbf{Y}}_{ni} } }$$

The deflection solution of the nanoplate ${\overline{\text{S}}}$-supported at *y* = 0 and *y* = *b*, denoted by $w_{{\text{S}}}^{{}} \left( {x,y} \right)$, is thus obtained as

41 $$w_{{\text{S}}}^{{}} \left( {x,y} \right) = \sum\limits_{n = 1,2,3, \ldots }^{\infty } {\sum\limits_{i = 1}^{4} {\sin } } \left( {\beta_{n} y} \right)X_{ni}^{{}} \left( x \right)$$

Substituting Eq. (41) into Eq. (24) to determine the constants, we obtain the deflection solution, $w_{1}^{{}} \left( {x,y} \right)$, of the first subproblem. For the uniform loading, we have

42 $$\begin{gathered} \frac{{w_{1}^{u} \left( {x,y} \right)}}{b} = \mathop \sum \limits_{n = 1}^{\infty } \frac{{2\overline{q}_{u} \left[ {\cos \left( {n\pi } \right) - 1} \right]\sin \left( {n\pi \overline{y}} \right)}}{{n\pi \psi_{n1} \psi_{n3} }}\left\{ {\left\{ {\cosh \left[ {{{\left( {2\overline{x} - 1} \right)\gamma_{n3} } \mathord{\left/ {\vphantom {{\left( {2\overline{x} - 1} \right)\gamma_{n3} } 2}} \right. \kern-\nulldelimiterspace} 2}} \right]{\text{sech}} \left( {{{\gamma_{n3} } \mathord{\left/ {\vphantom {{\gamma_{n3} } 2}} \right. \kern-\nulldelimiterspace} 2}} \right) - 1} \right\}\psi_{n1} } \right. \hfill \\ \left. { + \left\{ {\cosh \left[ {{{\left( {2\overline{x} - 1} \right)\gamma_{n1} } \mathord{\left/ {\vphantom {{\left( {2\overline{x} - 1} \right)\gamma_{n1} } 2}} \right. \kern-\nulldelimiterspace} 2}} \right]{\text{sech}} \left( {{{\gamma_{n1} } \mathord{\left/ {\vphantom {{\gamma_{n1} } 2}} \right. \kern-\nulldelimiterspace} 2}} \right) - 1} \right\}\psi_{n3} } \right\} \hfill \\ \end{gathered}$$

where $\phi = {b \mathord{\left/ {\vphantom {b a}} \right. \kern-\nulldelimiterspace} a}$, $\overline{q}_{u} = {{b^{3} q_{u} } \mathord{\left/ {\vphantom {{b^{3} q_{u} } D}} \right. \kern-\nulldelimiterspace} D}$, $\overline{x} = {x \mathord{\left/ {\vphantom {x a}} \right. \kern-\nulldelimiterspace} a}$, $\overline{y} = {y \mathord{\left/ {\vphantom {y b}} \right. \kern-\nulldelimiterspace} b}$, $\gamma_{n1} = a\xi_{n1}$, $\gamma_{n3} = a\xi_{n3}$, $\psi_{n1} = b{{^{4} \left[ {k_{w} - \overline{m}_{0} + \beta_{n}^{2} \left( {\beta_{n}^{2} \overline{R}_{y} + \tilde{R}_{y} } \right) - \overline{R}_{x} \xi_{n1}^{4} } \right]} \mathord{\left/ {\vphantom {{^{4} \left[ {k_{w} - \overline{m}_{0} + \beta_{n}^{2} \left( {\beta_{n}^{2} \overline{R}_{y} + \tilde{R}_{y} } \right) - \overline{R}_{x} \xi_{n1}^{4} } \right]} D}} \right. \kern-\nulldelimiterspace} D}$, and $\psi_{n3} = b{{^{4} \left[ {k_{w} - \overline{m}_{0} + \beta_{n}^{2} \left( {\beta_{n}^{2} \overline{R}_{y} + \tilde{R}_{y} } \right) - \overline{R}_{x} \xi_{n3}^{4} } \right]} \mathord{\left/ {\vphantom {{^{4} \left[ {k_{w} - \overline{m}_{0} + \beta_{n}^{2} \left( {\beta_{n}^{2} \overline{R}_{y} + \tilde{R}_{y} } \right) - \overline{R}_{x} \xi_{n3}^{4} } \right]} D}} \right. \kern-\nulldelimiterspace} D}$. For the concentrate loading, we have

43 $$\begin{gathered} \frac{{w_{1}^{c} \left( {x,y} \right)}}{b} = \mathop \sum \limits_{n = 1}^{\infty } \frac{{2\phi \overline{q}_{c} }}{{\psi_{n1} \psi_{n3} }}\sin \left( {n\pi \overline{y}} \right)\sin \left( {n\pi \overline{y}_{0} } \right)\left\{ {\left\{ {\gamma_{n3} \psi_{n1} \sinh \left[ {\gamma_{n3} \left( {\overline{x}_{0} - \overline{x}} \right)} \right] + \gamma_{n1} \psi_{n3} \sinh \left[ {\gamma_{n1} \left( {\overline{x}_{0} - \overline{x}} \right)} \right]} \right\}} \right.H\left( {\overline{x} - \overline{x}_{0} } \right) \hfill \\ \left. { + \gamma_{n3} \psi_{n1} {\text{csch}} \left( {\gamma_{n3} } \right)\sinh \left( {\gamma_{n3} \overline{x}} \right)\sinh \left[ {\gamma_{n3} \left( {1 - \overline{x}_{0} } \right)} \right] + \gamma_{n1} \psi_{n3} {\text{csch}} \left( {\gamma_{n1} } \right)\sinh \left( {\gamma_{n1} \overline{x}} \right)\sinh \left[ {\gamma_{n1} \left( {1 - \overline{x}_{0} } \right)} \right]} \right\} \hfill \\ \end{gathered}$$

where $$\overline{\phi } = {a \mathord{\left/ {\vphantom {a b}} \right. \kern-\nulldelimiterspace} b}$$, $$\overline{y}_{0} = {{y_{0} } \mathord{\left/ {\vphantom {{y_{0} } b}} \right. \kern-\nulldelimiterspace} b}$$, $$\overline{x}_{0} = {{x_{0} } \mathord{\left/ {\vphantom {{x_{0} } a}} \right. \kern-\nulldelimiterspace} a}$$, and $$\overline{q}_{c} = {{bq_{c} } \mathord{\left/ {\vphantom {{bq_{c} } D}} \right. \kern-\nulldelimiterspace} D}$$.

Equating $q_{c}$ or $q_{u}$ with zero, and imposing the BCs in Eq. (25), we obtain the deflection solution of the second subproblem, denoted by $w_{2}^{{}} \left( {x,y} \right)$, as

44 $$\begin{gathered} \frac{{w_{2} \left( {x,y} \right)}}{a} = \mathop \sum \limits_{n = 1,2,3, \ldots }^{\infty } \frac{{\sin \left( {n\pi \overline{y}} \right)}}{{\left( {\gamma_{n1}^{2} - \gamma_{n3}^{2} } \right)}}\left\{ {\left\{ {{\text{csch}} \left( {\gamma_{n1} } \right)\sinh \left[ {\gamma_{n1} \left( {\overline{x} - 1} \right)} \right] - {\text{csch}} \left( {\gamma_{n3} } \right)\sinh \left[ {\gamma_{n3} \left( {\overline{x} - 1} \right)} \right]} \right\}\overline{E}_{n} } \right. + \\ \left. {\left[ {{\text{csch}} \left( {\gamma_{n3} } \right)\sinh \left( {\gamma_{n3} \overline{x}} \right) - {\text{csch}} \left( {\gamma_{n1} } \right)\sinh \left( {\gamma_{n1} \overline{x}} \right)} \right]\overline{F}_{n} } \right\} \\ \end{gathered}$$

where $$\overline{E}_{n} = {{aE_{n} } \mathord{\left/ {\vphantom {{aE_{n} } {\overline{R}}}} \right. \kern-\nulldelimiterspace} {\overline{R}}}_{x}$$, and $$\overline{F}_{n} = {{aF_{n} } \mathord{\left/ {\vphantom {{aF_{n} } {\overline{R}}}} \right. \kern-\nulldelimiterspace} {\overline{R}}}_{x}$$.

For the third subproblem, incorporating Eq. (26), we obtain the deflection solution, denoted by $w_{3}^{{}} \left( {x,y} \right)$, following the second subproblem, i.e.,

45 $$\begin{gathered} \frac{{w_{3}^{{}} \left( {x,y} \right)}}{b} = \mathop \sum \limits_{n = 1}^{\infty } \frac{{\sin \left( {n\pi \overline{x}} \right)}}{{\left( {\overline{\gamma }_{n1}^{2} - \overline{\gamma }_{n3}^{2} } \right)}}\left\{ {\left\{ {{\text{csch}} \left( {\overline{\gamma }_{n1} } \right)\sinh \left[ {\overline{\gamma }_{n1} \left( {\overline{y} - 1} \right)} \right] - {\text{csch}} \left( {\overline{\gamma }_{n3} } \right)\sinh \left[ {\overline{\gamma }_{n3} \left( {\overline{y} - 1} \right)} \right]} \right\}\overline{G}_{n} } \right. \\ \left. { + \left[ {{\text{csch}} \left( {\overline{\gamma }_{n3} } \right)\sinh \left( {\overline{\gamma }_{n3} \overline{y}} \right) - {\text{csch}} \left( {\overline{\gamma }_{n1} } \right)\sinh \left( {\overline{\gamma }_{n1} \overline{y}} \right)} \right]\overline{H}_{n} } \right\} \\ \end{gathered}$$

where $$\overline{\gamma }_{n1} = b\overline{\xi }_{n1}$$, $$\overline{\gamma }_{n3} = b\overline{\xi }_{n3}$$, $$\overline{G}_{n} = {{bG_{n} } \mathord{\left/ {\vphantom {{bG_{n} } {\overline{R}}}} \right. \kern-\nulldelimiterspace} {\overline{R}}}_{y}$$, $$\overline{H}_{n} = {{bH_{n} } \mathord{\left/ {\vphantom {{bH_{n} } {\overline{R}}}} \right. \kern-\nulldelimiterspace} {\overline{R}}}_{y}$$,$$\overline{\xi }_{n1} = \sqrt {{{\left\{ {\tilde{R}_{y} + \overline{R}_{xy} \alpha_{n}^{2} - \sqrt {\left( {\overline{R}_{xy} \alpha_{n}^{2} + \tilde{R}_{y} } \right)^{2} - 4\overline{R}_{y} \left[ {\alpha_{n}^{2} \left( {\tilde{R}_{x} + \alpha_{n}^{2} \overline{R}_{x} } \right) + k_{w} - \overline{m}_{0} } \right]} } \right\}} \mathord{\left/ {\vphantom {{\left\{ {\tilde{R}_{y} + \overline{R}_{xy} \alpha_{n}^{2} - \sqrt {\left( {\overline{R}_{xy} \alpha_{n}^{2} + \tilde{R}_{y} } \right)^{2} - 4\overline{R}_{y} \left[ {\alpha_{n}^{2} \left( {\tilde{R}_{x} + \alpha_{n}^{2} \overline{R}_{x} } \right) + k_{w} - \overline{m}_{0} } \right]} } \right\}} {\left( {2\overline{R}_{y} } \right)}}} \right. \kern-\nulldelimiterspace} {\left( {2\overline{R}_{y} } \right)}}}$$, and $$\overline{\xi }_{n3} = \sqrt {{{\left\{ {\tilde{R}_{y} + \overline{R}_{xy} \alpha_{n}^{2} + \sqrt {\left( {\overline{R}_{xy} \alpha_{n}^{2} + \tilde{R}_{y} } \right)^{2} - 4\overline{R}_{y} \left[ {\alpha_{n}^{2} \left( {\tilde{R}_{x} + \alpha_{n}^{2} \overline{R}_{x} } \right) + k_{w} - \overline{m}_{0} } \right]} } \right\}} \mathord{\left/ {\vphantom {{\left\{ {\tilde{R}_{y} + \overline{R}_{xy} \alpha_{n}^{2} + \sqrt {\left( {\overline{R}_{xy} \alpha_{n}^{2} + \tilde{R}_{y} } \right)^{2} - 4\overline{R}_{y} \left[ {\alpha_{n}^{2} \left( {\tilde{R}_{x} + \alpha_{n}^{2} \overline{R}_{x} } \right) + k_{w} - \overline{m}_{0} } \right]} } \right\}} {\left( {2\overline{R}_{y} } \right)}}} \right. \kern-\nulldelimiterspace} {\left( {2\overline{R}_{y} } \right)}}}$$.

## Analytic solutions for rectangular nanoplates with combinations of clamped and simply supported edges

Superposing the fundamental analytic solutions given in “Fundamental analytic solutions in the symplectic space” section, analytic bending, buckling, and free vibration solutions of rectangular nanoplates with combinations of clamped and simply supported edges can be obtained, provided that the BCs are satisfied.

Denoting the clamped edge by “C”, a fully clamped (CCCC) nanoplate resting on an elastic foundation is first solved, where zero rotation conditions should be satisfied at each edge. Therefore, the following equations hold:

46 $$\sum\limits_{i = 1}^{3} {\left. {\frac{{\partial w_{i} }}{\partial x}} \right|}_{x = 0,a} = 0,\quad \sum\limits_{i = 1}^{3} {\left. {\frac{{\partial w_{i} }}{\partial y}} \right|}_{y = 0,b} = 0$$

For a uniformly loaded CCCC nanoplate, substituting Eqs. (42), (44), (45) into Eq. (46) and expanding the existed polynomials as sine series, using the orthogonality in the trigonometric series, we have

47 $$\begin{aligned} & \frac{{\gamma_{m1} \coth \left( {\gamma_{m1} } \right) - \gamma_{m3} \coth \left( {\gamma_{m3} } \right)}}{{\gamma_{m1}^{2} - \gamma_{m3}^{2} }}\overline{E}_{m} + \frac{{\gamma_{m3} {\text{csch}} \left( {\gamma_{m3} } \right) - \gamma_{m1} {\text{csch}} \left( {\gamma_{m1} } \right)}}{{\gamma_{m1}^{2} - \gamma_{m3}^{2} }}\overline{F}_{m} \\ & \quad \quad + \mathop \sum \limits_{n = 1,2,3, \ldots }^{\infty } \frac{{2\phi mn\pi^{2} }}{{\left( {m^{2} \pi^{2} + \overline{\gamma }_{n1}^{2} } \right)\left( {m^{2} \pi^{2} + \overline{\gamma }_{n3}^{2} } \right)}}\left[ {\overline{G}_{n} - \cos \left( {m\pi } \right)\overline{H}_{n} } \right] \\ & \quad = \frac{{2\phi \overline{q}_{u} \left[ {\cos \left( {m\pi } \right) - 1} \right]\left[ {\gamma_{m1} \psi_{m3} \tanh \left( {{{\gamma_{m1} } \mathord{\left/ {\vphantom {{\gamma_{m1} } 2}} \right. \kern-\nulldelimiterspace} 2}} \right) + \gamma_{m3} \psi_{m1} \tanh \left( {{{\gamma_{m3} } \mathord{\left/ {\vphantom {{\gamma_{m3} } 2}} \right. \kern-\nulldelimiterspace} 2}} \right)} \right]}}{{n\pi \psi_{m1} \psi_{m3} }} \\ \end{aligned}$$

for $$x = 0$$ ($$m = 1,2,3, \ldots$$),

48 $$\begin{aligned} & \frac{{\gamma_{m1} {\text{csch}} \left( {\gamma_{m1} } \right) - \gamma_{m3} {\text{csch}} \left( {\gamma_{m3} } \right)}}{{\gamma_{m1}^{2} - \gamma_{m3}^{2} }}\overline{E}_{m} + \frac{{\gamma_{m3} \coth \left( {\gamma_{m3} } \right) - \gamma_{m1} \coth \left( {\gamma_{m1} } \right)}}{{\gamma_{m1}^{2} - \gamma_{m3}^{2} }}\overline{F}_{m} \\ & \quad \quad + \mathop \sum \limits_{n = 1,2,3, \ldots }^{\infty } \frac{{2\phi mn\pi^{2} \cos \left( {n\pi } \right)}}{{\left( {m^{2} \pi^{2} + \overline{\gamma }_{n1}^{2} } \right)\left( {m^{2} \pi^{2} + \overline{\gamma }_{n3}^{2} } \right)}}\left[ {\overline{G}_{n} - \cos \left( {m\pi } \right)\overline{H}_{n} } \right] \\ & \quad = - \frac{{2\phi \overline{q}_{u} \left[ {\cos \left( {m\pi } \right) - 1} \right]\left[ {\gamma_{m1} \psi_{m3} \tanh \left( {{{\gamma_{m1} } \mathord{\left/ {\vphantom {{\gamma_{m1} } 2}} \right. \kern-\nulldelimiterspace} 2}} \right) + \gamma_{m3} \psi_{m1} \tanh \left( {{{\gamma_{m3} } \mathord{\left/ {\vphantom {{\gamma_{m3} } 2}} \right. \kern-\nulldelimiterspace} 2}} \right)} \right]}}{{n\pi \psi_{m1} \psi_{m3} }} \\ \end{aligned}$$

for $$x = a$$ ($$m = 1,2,3, \ldots$$),

49 $$\begin{aligned} & \frac{{\overline{\gamma }_{m1} \coth \left( {\overline{\gamma }_{m1} } \right) - \overline{\gamma }_{m3} \coth \left( {\overline{\gamma }_{m3} } \right)}}{{\overline{\gamma }_{m1}^{2} - \overline{\gamma }_{m3}^{2} }}\overline{G}_{m} + \frac{{\overline{\gamma }_{m3} {\text{csch}} \left( {\overline{\gamma }_{m3} } \right) - \overline{\gamma }_{m1} {\text{csch}} \left( {\overline{\gamma }_{m1} } \right)}}{{\overline{\gamma }_{m1}^{2} - \overline{\gamma }_{m3}^{2} }}\overline{H}_{m} \\ & \quad \quad + \mathop \sum \limits_{n = 1,2,3, \ldots }^{\infty } \frac{{2\overline{\phi }mn\pi^{2} }}{{\left( {m^{2} \pi^{2} + \gamma_{n1}^{2} } \right)\left( {m^{2} \pi^{2} + \gamma_{n3}^{2} } \right)}}\left[ {\overline{E}_{n} - \cos \left( {m\pi } \right)\overline{F}_{n} } \right] \\ & \quad = \sum\limits_{n = 1,2,3, \ldots }^{\infty } {\frac{{ - 16\overline{q}_{u} \sin \left( {{{m\pi } \mathord{\left/ {\vphantom {{m\pi } 2}} \right. \kern-\nulldelimiterspace} 2}} \right)^{2} \sin \left( {{{n\pi } \mathord{\left/ {\vphantom {{n\pi } 2}} \right. \kern-\nulldelimiterspace} 2}} \right)^{2} \left[ {\psi_{n1} \gamma_{n3}^{2} \left( {m^{2} \pi^{2} + \gamma_{n1}^{2} } \right) + \psi_{n3} \gamma_{n1}^{2} \left( {m^{2} \pi^{2} + \gamma_{n3}^{2} } \right)} \right]}}{{m\pi \psi_{n1} \psi_{n3} \left( {m^{2} \pi^{2} + \gamma_{n1}^{2} } \right)\left( {m^{2} \pi^{2} + \gamma_{n3}^{2} } \right)}}} \\ \end{aligned}$$

for $$y = 0$$ ($$m = 1,2,3, \ldots$$), and

50 $$\begin{gathered} \frac{{\overline{\gamma }_{m1} {\text{csch}} \left( {\overline{\gamma }_{m1} } \right) - \overline{\gamma }_{m3} {\text{csch}} \left( {\overline{\gamma }_{m3} } \right)}}{{\overline{\gamma }_{m1}^{2} - \overline{\gamma }_{m3}^{2} }}\overline{G}_{m} + \frac{{\overline{\gamma }_{m3} \coth \left( {\overline{\gamma }_{m3} } \right) - \overline{\gamma }_{m1} \coth \left( {\overline{\gamma }_{m1} } \right)}}{{\overline{\gamma }_{m1}^{2} - \overline{\gamma }_{m3}^{2} }}\overline{H}_{m} + \hfill \\ \mathop \sum \limits_{n = 1,2,3, \ldots }^{\infty } \frac{{2\overline{\phi }mn\pi^{2} \cos \left( {n\pi } \right)}}{{\left( {m^{2} \pi^{2} + \gamma_{n1}^{2} } \right)\left( {m^{2} \pi^{2} + \gamma_{n3}^{2} } \right)}}\left[ {\overline{E}_{n} - \cos \left( {m\pi } \right)\overline{F}_{n} } \right] \hfill \\ = \mathop \sum \limits_{n = 1,2,3, \ldots }^{\infty } \frac{{ - 16\overline{q}_{u} \cos \left( {n\pi } \right)\sin \left( {{{m\pi } \mathord{\left/ {\vphantom {{m\pi } 2}} \right. \kern-\nulldelimiterspace} 2}} \right)^{2} \sin \left( {{{n\pi } \mathord{\left/ {\vphantom {{n\pi } 2}} \right. \kern-\nulldelimiterspace} 2}} \right)^{2} \left[ {\psi_{n1} \gamma_{n3}^{2} \left( {m^{2} \pi^{2} + \gamma_{n1}^{2} } \right) + \psi_{n3} \gamma_{n1}^{2} \left( {m^{2} \pi^{2} + \gamma_{n3}^{2} } \right)} \right]}}{{m\pi \psi_{n1} \psi_{n3} \left( {m^{2} \pi^{2} + \gamma_{n1}^{2} } \right)\left( {m^{2} \pi^{2} + \gamma_{n3}^{2} } \right)}} \hfill \\ \end{gathered}$$

for $$y = b$$ ($$m = 1,2,3, \ldots$$).

For a CCCC nanoplate under concentrated load, the only differences are on the right-hand sides of Eqs. (47–50), which become

51 $$\frac{{2\phi^{2} \overline{q}_{c} \sin \left( {m\pi \overline{y}_{0} } \right)}}{{\psi_{m1} \psi_{m3} }}\left\{ {\psi_{m1} \gamma_{m3}^{2} {\text{csch}} \left( {\gamma_{m3} } \right)\sinh \left[ {\gamma_{m3} \left( {\overline{x}_{0} - 1} \right)} \right] + \psi_{m3} \gamma_{m1}^{2} {\text{csch}} \left( {\gamma_{m1} } \right)\sinh \left[ {\gamma_{m1} \left( {\overline{x}_{0} - 1} \right)} \right]} \right\}$$

52 $$\frac{{2\phi^{2} \overline{q}_{c} \sin \left( {m\pi \overline{y}_{0} } \right)}}{{\psi_{m1} \psi_{m3} }}\left[ {\psi_{m1} \gamma_{m3}^{2} {\text{csch}} \left( {\gamma_{m3} } \right)\sinh \left( {\gamma_{m3} \overline{x}_{0} } \right) + \psi_{m3} \gamma_{m1}^{2} {\text{csch}} \left( {\gamma_{m1} } \right)\sinh \left( {\gamma_{m1} \overline{x}_{0} } \right)} \right]$$

53 $$- \sum\limits_{n = 1,2,3, \ldots }^{\infty } {\frac{{4n\pi \phi \overline{q}_{c} \sin \left( {m\pi \overline{x}_{0} } \right)\sin \left( {n\pi \overline{y}_{0} } \right)\left[ {\psi_{n1} \gamma_{n3}^{2} \left( {m^{2} \pi^{2} + \gamma_{n1}^{2} } \right) + \psi_{n3} \gamma_{n1}^{2} \left( {m^{2} \pi^{2} + \gamma_{n3}^{2} } \right)} \right]}}{{\psi_{n1} \psi_{n3} \left( {m^{2} \pi^{2} + \gamma_{n1}^{2} } \right)\left( {m^{2} \pi^{2} + \gamma_{n3}^{2} } \right)}}}$$

54 $$- \sum\limits_{n = 1,2,3, \ldots }^{\infty } {\frac{{4n\pi \phi \overline{q}_{c} \cos \left( {n\pi } \right)\sin \left( {m\pi \overline{x}_{0} } \right)\sin \left( {n\pi \overline{y}_{0} } \right)\left[ {\psi_{n1} \gamma_{n3}^{2} \left( {m^{2} \pi^{2} + \gamma_{n1}^{2} } \right) + \psi_{n3} \gamma_{n1}^{2} \left( {m^{2} \pi^{2} + \gamma_{n3}^{2} } \right)} \right]}}{{\psi_{n1} \psi_{n3} \left( {m^{2} \pi^{2} + \gamma_{n1}^{2} } \right)\left( {m^{2} \pi^{2} + \gamma_{n3}^{2} } \right)}}}$$

for Eqs. (47–50), respectively.

For the bending problem, equating $N_{x}$, $N_{y}$, and $\omega$ with zero, the constants $\overline{E}_{n}$, $\overline{F}_{n}$, $\overline{G}_{n}$, and $\overline{H}_{n}$ ($n = 1,2,3, \ldots$) are obtained by solving the nonhomogeneous equations (47–50) for the case of uniform load or incorporating Eqs. (51–54) for the case of concentrated load. Substituting the constants into Eqs. (44) and (45), followed by summation of Eqs. (42)/(43), (44), and (45), the final bending solution is obtained. For the buckling (free vibration) problem, equating $q_{u}$, $q_{c}$, $N_{x}$, $N_{y}$, $k_{w}$, and $k_{p}$ ($q_{u}$, $q_{c}$, $\omega$, $k_{w}$, and $k_{p}$) with zero, the buckling loads (natural frequencies) are determined by equating with zero the determinant of the coefficient matrix of the homogeneous simultaneous equations of Eqs. (47–50). Substituting the nonzero constant solutions into the Eqs. (44) and (45), and conducting summation, the buckling (vibration) mode shapes are obtained.

For the nanoplates with any other combinations of clamped and simply supported edges, the solutions can be obtained by relaxation of BCs from the above derivations. By equating $N_{x}$, $N_{y}$, and $\omega$ with zero, imposing $H_{n} = 0$ or $H_{n} = - {{2\mu q_{u} \left[ {\cos \left( {n\pi } \right) - 1} \right]} \mathord{\left/ {\vphantom {{2\mu q_{u} \left[ {\cos \left( {n\pi } \right) - 1} \right]} {\left( {n\pi } \right)}}} \right. \kern-\nulldelimiterspace} {\left( {n\pi } \right)}}$ ($n = 1,2,3, \ldots$), and eliminating Eq. (50), we have three sets of simultaneous linear equations for the bending solutions of a CCCS nanoplate under concentrated or uniform loads. By imposing $F_{n} = H_{n} = 0$ or $F_{n} = H_{n} = - {{2\mu q_{u} \left[ {\cos \left( {n\pi } \right) - 1} \right]} \mathord{\left/ {\vphantom {{2\mu q_{u} \left[ {\cos \left( {n\pi } \right) - 1} \right]} {\left( {n\pi } \right)}}} \right. \kern-\nulldelimiterspace} {\left( {n\pi } \right)}}$ ($n = 1,2,3, \ldots$), and eliminating Eqs. (48) and (50), we obtain the bending solutions of a CCSS nanoplate under concentrated or uniform loads. Similar treatments yield the bending solutions of SCSC, SCSS, and SSSS nanoplates. Here, an anti-clockwise four-letter notation, starting from the edge $x = 0$, has been used to label a nanoplate under different BCs. The buckling (free vibration) solutions are obtained in a similar way after equating $q_{u}$, $q_{c}$, $\omega$, $k_{w}$ and $k_{p}$ ($q_{u}$, $q_{c}$, $N_{x}$, $N_{y}$, $k_{w}$ and $k_{p}$) with zero.

## Comprehensive numerical results and discussion

Comprehensive numerical results of the nanoplates with combinations of clamped and simply supported edges are presented to confirm the validity of the developed method, and, more importantly, to provide benchmark solutions for future comparison.

The convergence study is carried out and the results are shown in Table 1 for the square nanoplates with *b* = 10 nm, $\mu = 1\;{\text{nm}}^{2}$, and $\overline{k}_{p} = {{k_{p} b^{2} } \mathord{\left/ {\vphantom {{k_{p} b^{2} } D}} \right. \kern-\nulldelimiterspace} D} =$ 20 under different BCs, including the central bending deflections, ${{10^{5} Dw\left( {{a \mathord{\left/ {\vphantom {a {2,{b \mathord{\left/ {\vphantom {b 2}} \right. \kern-\nulldelimiterspace} 2}}}} \right. \kern-\nulldelimiterspace} {2,{b \mathord{\left/ {\vphantom {b 2}} \right. \kern-\nulldelimiterspace} 2}}}} \right)} \mathord{\left/ {\vphantom {{10^{5} Dw\left( {{a \mathord{\left/ {\vphantom {a {2,{b \mathord{\left/ {\vphantom {b 2}} \right. \kern-\nulldelimiterspace} 2}}}} \right. \kern-\nulldelimiterspace} {2,{b \mathord{\left/ {\vphantom {b 2}} \right. \kern-\nulldelimiterspace} 2}}}} \right)} {\left( {q_{u} b^{4} } \right)}}} \right. \kern-\nulldelimiterspace} {\left( {q_{u} b^{4} } \right)}}$ and ${{10^{5} Dw\left( {{a \mathord{\left/ {\vphantom {a {2,{b \mathord{\left/ {\vphantom {b 2}} \right. \kern-\nulldelimiterspace} 2}}}} \right. \kern-\nulldelimiterspace} {2,{b \mathord{\left/ {\vphantom {b 2}} \right. \kern-\nulldelimiterspace} 2}}}} \right)} \mathord{\left/ {\vphantom {{10^{5} Dw\left( {{a \mathord{\left/ {\vphantom {a {2,{b \mathord{\left/ {\vphantom {b 2}} \right. \kern-\nulldelimiterspace} 2}}}} \right. \kern-\nulldelimiterspace} {2,{b \mathord{\left/ {\vphantom {b 2}} \right. \kern-\nulldelimiterspace} 2}}}} \right)} {\left( {q_{c} b^{2} } \right)}}} \right. \kern-\nulldelimiterspace} {\left( {q_{c} b^{2} } \right)}}$, critical buckling load factors, ${{ - N_{x}^{{{\text{critical}}}} b^{2} } \mathord{\left/ {\vphantom {{ - N_{x}^{{{\text{critical}}}} b^{2} } D}} \right. \kern-\nulldelimiterspace} D}$, and fundamental frequency parameters, $\omega b^{2} \sqrt {{{\rho h} \mathord{\left/ {\vphantom {{\rho h} D}} \right. \kern-\nulldelimiterspace} D}}$, where the convergent results with the accuracy of five significant figures are marked in bold. It is found that only 10 terms, at most, yield the convergence to the last digit of five significant figures for the buckling and free vibration solutions in this study, but 80 and 320 terms, at most, are needed to achieve the same accuracy for the bending solutions with uniform and concentrated loads, respectively. In Table 2, the present buckling and free vibration solutions are compared with their counterparts available in the literature by molecular dynamics simulation^2,3^, Rayleigh–Ritz method^28^, and iSOV method^31^, respectively, confirming the validity of the adopted nonlocal theory and the present method. The parameters adopted are as follows^2,3,53,54^: $\rho = 2250\,{\text{kg}}/ { {\text{m}}^{3}}$, $E = 1\,{\text{TPa}}$, $\nu = 0.16$, and $h = 0.34\,{\text{nm}}$. It should be noted that Young’s moduli may be significantly different in the directions along short and long sides of a rectangular nanoplate^55,56^, which is not considered for a 5 nm × 2.5 nm nanoplate in reference^28^ and thus in the present study for comparison purpose.

**Table 1:** Convergence study for bending, buckling, and free vibration solutions of the square nanoplates with *b* = 10 nm, $\mu = 1\;{\text{nm}}^{2}$, and $\overline{k}_{p} =$ 20 under different BCs.

Mechanical quantity	Number of series terms	BC					
		CCCC	CCCS	CCSS	SCSC	SCSS	SSSS
${{10^{5} Dw\left( {{a \mathord{\left/ {\vphantom {a {2,{b \mathord{\left/ {\vphantom {b 2}} \right. \kern-\nulldelimiterspace} 2}}}} \right. \kern-\nulldelimiterspace} {2,{b \mathord{\left/ {\vphantom {b 2}} \right. \kern-\nulldelimiterspace} 2}}}} \right)} \mathord{\left/ {\vphantom {{10^{5} Dw\left( {{a \mathord{\left/ {\vphantom {a {2,{b \mathord{\left/ {\vphantom {b 2}} \right. \kern-\nulldelimiterspace} 2}}}} \right. \kern-\nulldelimiterspace} {2,{b \mathord{\left/ {\vphantom {b 2}} \right. \kern-\nulldelimiterspace} 2}}}} \right)} {\left( {q_{u} b^{4} } \right)}}} \right. \kern-\nulldelimiterspace} {\left( {q_{u} b^{4} } \right)}}$	10	70.575	85.081	106.25	100.52	129.81	163.76
	20	70.532	85.028	106.22	100.51	129.81	163.76
	40	70.527	85.022	106.22	100.51	129.81	163.76
	**80**	**70.526**	**85.021**	**106.22**	**100.51**	**129.81**	**163.76**
	120	70.526	85.021	106.216	100.510	129.810	163.760
${{10^{5} Dw\left( {{a \mathord{\left/ {\vphantom {a {2,{b \mathord{\left/ {\vphantom {b 2}} \right. \kern-\nulldelimiterspace} 2}}}} \right. \kern-\nulldelimiterspace} {2,{b \mathord{\left/ {\vphantom {b 2}} \right. \kern-\nulldelimiterspace} 2}}}} \right)} \mathord{\left/ {\vphantom {{10^{5} Dw\left( {{a \mathord{\left/ {\vphantom {a {2,{b \mathord{\left/ {\vphantom {b 2}} \right. \kern-\nulldelimiterspace} 2}}}} \right. \kern-\nulldelimiterspace} {2,{b \mathord{\left/ {\vphantom {b 2}} \right. \kern-\nulldelimiterspace} 2}}}} \right)} {\left( {q_{c} b^{2} } \right)}}} \right. \kern-\nulldelimiterspace} {\left( {q_{c} b^{2} } \right)}}$	40	330.37	351.37	380.94	373.81	413.82	460.12
	80	330.52	351.53	381.10	373.97	413.98	460.28
	160	330.55	351.56	381.14	374.01	414.02	460.32
	240	330.56	351.57	381.14	374.02	414.02	460.33
	**320**	**330.57**	**351.57**	**381.15**	**374.02**	**414.03**	**460.33**
	400	330.57	351.57	381.15	374.02	414.03	460.33
${{ - N_{x}^{{{\text{critical}}}} b^{2} } \mathord{\left/ {\vphantom {{ - N_{x}^{{{\text{critical}}}} b^{2} } D}} \right. \kern-\nulldelimiterspace} D}$	3	34.352	29.844	24.271	–	–	–
	5	34.359	29.848	24.272	–	–	–
	10	**34.359**	**29.848**	**24.273**	–	–	–
	20	34.359	29.848	24.273	–	–	–
$\omega b^{2} \sqrt {{{\rho h} \mathord{\left/ {\vphantom {{\rho h} D}} \right. \kern-\nulldelimiterspace} D}}$	3	32.240	28.620	24.418	–	–	–
	5	32.251	28.626	24.421	–	–	–
	**10**	**32.252**	**28.626**	**24.421**	–	–	–
	20	32.252	28.626	24.421	–	–	–

**Table 2:** Comprehensive comparison of the critical buckling load and fundamental frequency solutions.

Buckling	BC	Critical buckling load (N/m)	*a* = *b* (nm)					
			4.99	8.08	10.77	14.65	18.51	22.35
	SSSS	Present (*μ* = 1.84 nm^2^)	1.08381	0.653020	0.435623	0.264407	0.175096	0.123826
		Molecular dynamics^3^	1.0837	0.6536	0.4331	0.2609	0.1714	0.1191

		$- N_{x}^{{{\text{crtical}}}} b^{2} /D$ $(N_{y} /N_{x} = 1, a = b = 5 {\text{nm}}$)	*μ* (nm^2^)					
			0	1	2	4		
	CCCC	Present	52.3447	16.9193	10.0904	5.58334		
		iSOV^31^	52.4550	16.9308	–	5.5846		
	CCSS	Present	32.0524	14.0452	8.99289	5.23016		
		iSOV^31^	32.0859	14.0516	–	5.2310		
	SCSC	Present	37.7996	15.0477	9.39361	5.36322		
		iSOV^31^	37.7996	15.0477	9.3936	5.3632		
	SCSS	Present	26.2798	12.8120	8.47084	5.04918		
		iSOV^31^	26.2798	12.8120	8.4708	5.0492		
	SSSS	Present	19.7392	11.0302	7.65342	4.74697		
		iSOV^31^	19.7392	11.0302	–	4.7470		

Vibration	BC	Fundamental frequency (THz)	*a* = *b* (nm)					
			10	15	20	25	30	35
	CCCC	Present (*μ* = 0.28 nm^2^)	0.166204	0.0525765	0.0297640	0.0191059	0.0132896	0.00977343
		Molecular dynamics^2^	0.1162438	0.0534719	0.0307422	0.0180318	0.0133060	0.0104205
	SSSS	Present (*μ* = 1.30 nm^2^)	0.0586902	0.0277179	0.0159556	0.0103251	0.00721415	0.00531994
		Molecular dynamics^2^	0.0587725	0.0273881	0.0157524	0.0099840	0.0070655	0.0052982

		$\omega a^{2} \sqrt {\rho h/D}$	Mode (*a* = 5 nm, *b* = 2.5 nm, *μ* = 2 nm^2^)					
			1st	2nd	3rd	4th		
	CCCC	Present	40.2855	43.4400	50.1684	58.9583		
		Rayleigh–Ritz method^28^	40.2856	43.4408	50.1693	58.9610		
	CCCS	Present	30.8646	37.5974	46.7039	53.5255		
		Rayleigh–Ritz method^28^	30.8647	37.5978	46.7047	53.5259		
	CCSS	Present	30.0688	35.5232	43.6944	53.1077		
		Rayleigh–Ritz method^28^	30.0688	35.5235	43.6951	53.1091		
	SCSC	Present	39.6000	40.7306	45.2329	52.4998		
		Rayleigh–Ritz method^28^	39.6000	40.7306	45.2329	52.5002		
	SCSS	Present	29.5537	33.8697	41.0402	49.9349		
		Rayleigh–Ritz method^28^	29.5537	33.8697	41.0402	49.9379		
	SSSS	Present	22.1851	29.1902	38.2287	44.1800		
		Rayleigh–Ritz method^28^	22.1851	29.1902	38.2287	44.1800		

To provide more comprehensive benchmark solutions, we have tabulated the bending, buckling, and free vibration solutions of CCCC, CCCS, CCSS, SCSC, SCSS, and SSSS nanoplates in Tables 3, 4, 5 and 6, with a total of 3000 numerical results presented. The central bending deflections, ${{10^{5} Dw\left( {{a \mathord{\left/ {\vphantom {a {2,{b \mathord{\left/ {\vphantom {b 2}} \right. \kern-\nulldelimiterspace} 2}}}} \right. \kern-\nulldelimiterspace} {2,{b \mathord{\left/ {\vphantom {b 2}} \right. \kern-\nulldelimiterspace} 2}}}} \right)} \mathord{\left/ {\vphantom {{10^{5} Dw\left( {{a \mathord{\left/ {\vphantom {a {2,{b \mathord{\left/ {\vphantom {b 2}} \right. \kern-\nulldelimiterspace} 2}}}} \right. \kern-\nulldelimiterspace} {2,{b \mathord{\left/ {\vphantom {b 2}} \right. \kern-\nulldelimiterspace} 2}}}} \right)} {\left( {q_{u} b^{4} } \right)}}} \right. \kern-\nulldelimiterspace} {\left( {q_{u} b^{4} } \right)}}$ and ${{10^{5} Dw\left( {{a \mathord{\left/ {\vphantom {a {2,{b \mathord{\left/ {\vphantom {b 2}} \right. \kern-\nulldelimiterspace} 2}}}} \right. \kern-\nulldelimiterspace} {2,{b \mathord{\left/ {\vphantom {b 2}} \right. \kern-\nulldelimiterspace} 2}}}} \right)} \mathord{\left/ {\vphantom {{10^{5} Dw\left( {{a \mathord{\left/ {\vphantom {a {2,{b \mathord{\left/ {\vphantom {b 2}} \right. \kern-\nulldelimiterspace} 2}}}} \right. \kern-\nulldelimiterspace} {2,{b \mathord{\left/ {\vphantom {b 2}} \right. \kern-\nulldelimiterspace} 2}}}} \right)} {\left( {q_{c} b^{2} } \right)}}} \right. \kern-\nulldelimiterspace} {\left( {q_{c} b^{2} } \right)}}$, of the six types of square nanoplates with *b* = 10 nm are tabulated in Tables 3 and 4 for the cases of uniform and concentrated loads, respectively, with $\overline{k}_{w} = {{k_{w} b^{4} } \mathord{\left/ {\vphantom {{k_{w} b^{4} } D}} \right. \kern-\nulldelimiterspace} D} = 200$, $\overline{k}_{p} = {{k_{p} b^{2} } \mathord{\left/ {\vphantom {{k_{p} b^{2} } D}} \right. \kern-\nulldelimiterspace} D} =$ 0, 5, 10, 15, and 20. The critical buckling load factors are tabulated in Table 5 for ${{N_{y} } \mathord{\left/ {\vphantom {{N_{y} } {N_{x} }}} \right. \kern-\nulldelimiterspace} {N_{x} }} =$ 1, 2, 3, 4, and 5. The frequency parameters are tabulated in Table 6 for the first five modes. In each of Tables 3, 4, 5 and 6, five equi-different nonlocal parameters are examined, i.e., $\mu = 0,\;1,\;2,\;3,\;{\text{and}}\;4\;{\text{nm}}^{2}$. The results by ABAQUS software^57^ based on the FEM are also presented in each table, which correspond to the classical thin plate theory and are valid for the cases with $\mu = 0$. In ABAQUS, the thickness-to-width ratio of the nanoplates is $10^{ - 3}$, and the S4R thin shell element with uniform size of *b*/200 is taken. Satisfactory agreement between the present solutions and their counterparts by the FEM is observed, further validating the present method.

**Table 3:** Bending deflections, ${{10^{5} Dw\left( {{a \mathord{\left/ {\vphantom {a {2,{b \mathord{\left/ {\vphantom {b 2}} \right. \kern-\nulldelimiterspace} 2}}}} \right. \kern-\nulldelimiterspace} {2,{b \mathord{\left/ {\vphantom {b 2}} \right. \kern-\nulldelimiterspace} 2}}}} \right)} \mathord{\left/ {\vphantom {{10^{5} Dw\left( {{a \mathord{\left/ {\vphantom {a {2,{b \mathord{\left/ {\vphantom {b 2}} \right. \kern-\nulldelimiterspace} 2}}}} \right. \kern-\nulldelimiterspace} {2,{b \mathord{\left/ {\vphantom {b 2}} \right. \kern-\nulldelimiterspace} 2}}}} \right)} {\left( {q_{u} b^{4} } \right)}}} \right. \kern-\nulldelimiterspace} {\left( {q_{u} b^{4} } \right)}}$, of uniformly loaded square nanoplates with *b* = 10 nm under different BCs.

*µ*	$\overline{k}_{p}$		BC					
			CCCC	CCCS	CCSS	SCSC	SCSS	SSSS
0	0	Present	108.83	130.23	164.08	153.39	203.31	265.33
		FEM	108.84	130.23	164.08	153.39	203.32	265.33
	5		100.36	118.60	146.47	138.23	178.21	226.39
	10		93.100	108.88	132.29	125.77	158.60	197.29
	15		86.822	100.63	120.63	115.36	142.87	174.74
	20		81.336	93.543	110.86	106.53	129.96	156.76
1	0		105.28	130.87	171.41	158.50	218.05	291.07
	5		93.726	115.32	148.59	138.54	186.41	243.89
	10		84.462	103.07	131.14	123.03	162.77	209.77
	15		76.8665	93.178	117.37	110.64	144.44	183.95
	20		70.526	85.021	106.22	100.51	129.81	163.76
2	0		101.94	131.38	177.84	163.09	230.94	313.20
	5		87.920	112.43	150.33	138.78	193.35	258.66
	10		77.291	98.25	130.20	120.77	166.27	220.23
	15		68.958	87.261	114.84	106.90	145.85	191.72
	20		62.249	78.482	102.72	95.890	129.89	169.72
3	0		98.816	131.78	183.50	167.22	242.26	332.34
	5		82.791	109.86	151.78	138.96	199.28	271.27
	10		71.243	94.198	129.42	118.88	169.26	229.14
	15		62.524	82.448	112.81	103.88	147.10	198.33
	20		55.708	73.306	99.983	92.234	130.07	174.82
4	0		95.873	132.09	188.51	170.94	252.27	349.00
	5		78.229	107.57	153.00	139.11	204.42	282.17
	10		66.073	90.733	128.76	117.28	171.84	236.81
	15		57.188	78.456	111.16	101.37	148.22	204.02
	20		50.411	69.106	97.789	89.266	130.31	179.21

**Table 4:** Bending deflections, ${{10^{5} Dw\left( {{a \mathord{\left/ {\vphantom {a {2,{b \mathord{\left/ {\vphantom {b 2}} \right. \kern-\nulldelimiterspace} 2}}}} \right. \kern-\nulldelimiterspace} {2,{b \mathord{\left/ {\vphantom {b 2}} \right. \kern-\nulldelimiterspace} 2}}}} \right)} \mathord{\left/ {\vphantom {{10^{5} Dw\left( {{a \mathord{\left/ {\vphantom {a {2,{b \mathord{\left/ {\vphantom {b 2}} \right. \kern-\nulldelimiterspace} 2}}}} \right. \kern-\nulldelimiterspace} {2,{b \mathord{\left/ {\vphantom {b 2}} \right. \kern-\nulldelimiterspace} 2}}}} \right)} {\left( {q_{c} b^{2} } \right)}}} \right. \kern-\nulldelimiterspace} {\left( {q_{c} b^{2} } \right)}}$, of centrally concentrate-loaded square nanoplates with *b* = 10 nm under different BCs.

*µ*	$\overline{k}_{p}$		BC					
			CCCC	CCCS	CCSS	SCSC	SCSS	SSSS
0	0	Present	497.93	541.68	609.34	589.07	687.68	810.08
		FEM	497.92	541.68	609.34	589.06	687.69	810.10
	5		462.99	499.37	553.69	538.53	615.51	708.21
	10		432.95	463.69	508.30	496.63	558.39	631.11
	15		432.95	433.18	470.48	461.29	511.94	570.59
	20		406.83	406.75	438.44	431.05	473.37	521.70
1	0		483.29	523.84	585.63	567.65	656.63	765.62
	5		432.96	466.24	515.57	502.04	571.59	655.00
	10		392.31	420.31	460.98	450.37	506.74	573.33
	15		358.75	382.81	417.15	408.56	455.54	510.40
	20		330.57	351.57	381.15	374.02	414.03	460.33
2	0		469.55	507.24	563.90	547.87	628.56	726.26
	5		406.61	437.25	482.40	470.21	533.58	609.31
	10		358.64	384.36	421.72	411.99	463.85	525.25
	15		320.85	342.96	374.72	366.70	410.41	461.82
	20		290.29	309.66	337.23	330.43	368.11	412.20
3	0		456.62	491.76	543.90	529.54	603.05	691.14
	5		383.30	411.67	453.28	442.20	500.35	569.64
	10		330.30	354.07	388.63	379.65	427.65	484.61
	15		290.20	310.64	340.16	332.63	373.45	421.74
	20		258.79	276.71	302.46	295.99	331.47	373.35
4	0		444.44	477.27	525.42	512.50	579.76	659.60
	5		362.52	388.94	427.49	417.35	471.05	534.86
	10		306.11	328.21	360.35	352.01	396.69	449.81
	15		264.90	283.90	311.45	304.37	342.62	388.11
	20		233.47	250.13	274.24	268.09	301.53	341.30

**Table 5:** Critical buckling load factors, ${{ - N_{x}^{{{\text{critical}}}} b^{2} } \mathord{\left/ {\vphantom {{ - N_{x}^{{{\text{critical}}}} b^{2} } D}} \right. \kern-\nulldelimiterspace} D}$, of square nanoplates with *b* = 10 nm under different BCs.

*µ*	$N_{y} /N_{x}$		BC					
			CCCC	CCCS	CCSS	SCSC	SCSS	SSSS
0	1	Present	52.345	42.547	32.052	37.800	26.280	19.739
		FEM	52.352	42.552	32.055	37.802	26.280	19.740
	2	Present	34.696	27.973	21.274	26.231	17.022	13.159
		FEM	34.700	27.975	21.275	26.232	17.023	13.158
	3	Present	25.835	20.762	15.865	20.062	12.573	9.8696
		FEM	25.837	20.764	15.866	20.064	12.573	9.8697
	4	Present	20.551	16.490	12.634	16.236	9.9633	7.8957
		FEM	20.553	16.492	12.635	16.238	9.9637	7.8960
	5	Present	17.051	13.671	10.491	13.634	8.2493	6.5798
		FEM	17.053	13.672	10.491	13.635	8.2497	6.5800
1	1		34.359	29.848	24.273	27.431	20.811	16.485
	2		22.754	19.206	16.114	19.712	13.334	10.990
	3		16.897	14.113	12.014	14.950	9.7984	8.2426
	4		13.393	11.143	9.5606	11.522	7.7414	6.5941
	5		11.072	9.2013	7.9315	9.3723	6.3968	5.4951
2	1		25.573	22.987	19.532	21.526	17.226	14.152
	2		16.899	14.621	12.966	15.689	10.959	9.4348
	3		12.419	10.682	9.6535	11.064	8.0265	7.0761
	4		9.4884	8.4014	7.6609	8.5450	6.3287	5.6609
	5		7.6762	6.9171	6.3334	6.9602	5.2222	4.7174
3	1		20.365	18.690	16.340	17.713	14.695	12.398
	2		13.396	11.801	10.843	12.425	9.3021	8.2651
	3		9.3726	8.5828	8.0431	8.7814	6.7967	6.1988
	4		7.1582	6.7213	6.3292	7.0569	5.3508	4.9590
	5		5.7892	5.5094	5.1820	5.5352	4.4103	4.1325
4	1		16.919	15.747	14.045	15.048	12.812	11.030
	2		10.892	9.8902	9.3122	10.2843	8.0803	7.3534
	3		7.5223	7.2448	6.8397	7.2794	5.8929	5.5151
	4		5.7400	5.5132	5.2966	5.6334	4.6329	4.4121
	5		4.6375	4.4494	4.3002	4.5494	3.8140	3.6767

**Table 6:** Frequency parameters, $\omega b^{2} \sqrt {{{\rho h} \mathord{\left/ {\vphantom {{\rho h} D}} \right. \kern-\nulldelimiterspace} D}}$, of square nanoplates with *b* = 10 nm under different BCs.

*µ*	Mode		BC					
			CCCC	CCCS	CCSS	SCSC	SCSS	SSSS
0	1	Present	35.985	31.826	27.054	28.951	23.646	19.739
		FEM	35.987	31.826	27.053	28.950	23.644	19.736
	2	Present	73.394	63.331	60.539	54.743	51.674	49.348
		FEM	73.404	63.335	60.542	54.744	51.673	49.346
	3	Present	73.394	71.076	60.786	69.327	58.646	78.957
		FEM	73.404	71.085	60.789	69.335	58.649	78.947
	4	Present	108.22	100.79	92.836	94.585	86.135	98.696
		FEM	108.23	100.80	92.836	94.587	86.130	98.711
	5	Present	131.58	116.36	114.56	102.22	100.27	128.31
		FEM	131.62	116.38	114.58	102.23	100.29	128.30
1	1		32.252	28.626	24.421	26.184	21.464	18.022
	2		58.273	50.808	48.654	44.419	42.043	40.285
	3		58.273	56.578	48.938	55.357	47.328	58.799
	4		78.048	73.237	67.969	69.294	63.595	69.687
	5		89.939	80.751	79.596	71.910	70.663	84.395
2	1		29.489	26.239	22.441	24.097	19.804	16.698
	2		49.807	43.664	41.840	38.389	36.385	34.926
	3		49.807	48.421	42.143	47.451	40.797	34.926
	4		64.186	60.407	56.231	57.341	52.791	48.979
	5		72.691	65.641	64.716	58.702	57.719	56.961
3	1		27.324	24.357	20.872	22.438	18.476	15.629
	2		44.192	38.874	37.259	34.292	32.530	31.259
	3		44.192	42.994	37.571	42.176	36.390	31.259
	4		55.773	52.574	49.020	49.994	46.105	42.856
	5		62.625	56.729	55.930	50.831	49.996	49.357
4	1		25.571	22.827	19.589	21.079	17.382	14.742
	2		40.122	35.378	33.911	31.277	29.686	28.548
	3		40.122	39.055	34.226	38.338	33.159	28.548
	4		49.981	47.162	44.018	44.896	41.449	38.573
	5		55.836	50.682	49.965	45.461	44.722	44.161

**Figure 2:**
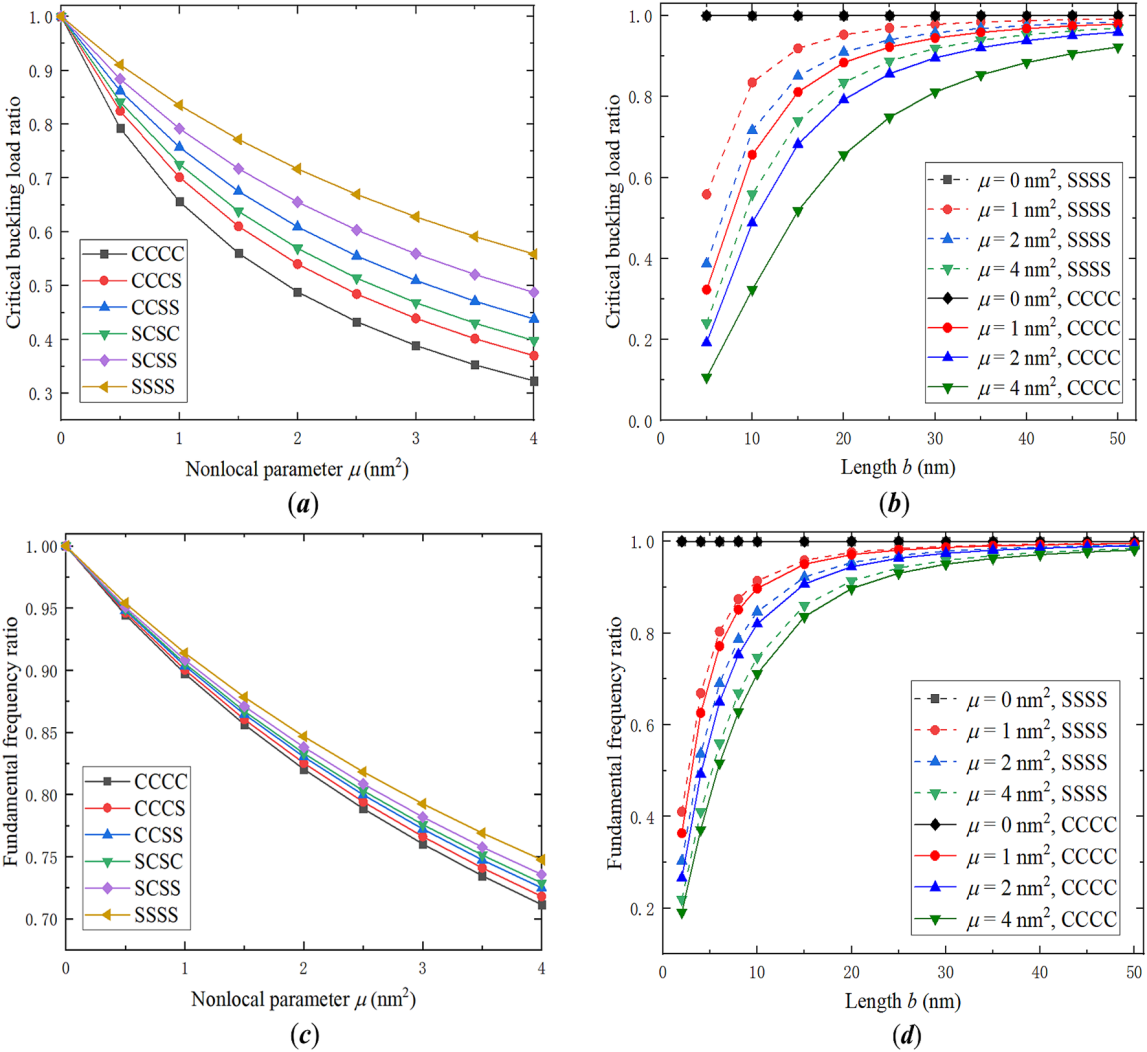
(**a**) Critical buckling load ratios versus the nonlocal parameter of square nanoplates under different BCs. (**b**) Critical buckling load ratios versus the length of square SSSS and CCCC nanoplates with different nonlocal parameters. (**c**) Fundamental frequency ratios versus the nonlocal parameter of square nanoplates under different BCs. (**d**) Fundamental frequency ratios versus the length of square SSSS and CCCC nanoplates with different nonlocal parameters.

Figuring out the effects of the nonlocal parameter and others on the mechanical behavior of a nanoplate is helpful for researchers to determine the nonlocal parameter and to design the structure^2,3,58–61^. Accordingly, quantitative parameter analyses are implemented with the analytic solutions obtained in this study. Defining the critical buckling load ratio as the ratio of the nonlocal theory- to classical theory-based critical buckling loads, Fig. 2a plots the nonlocal parameter dependent ratios of the six types of square nanoplates with $b = 10\,{\text{nm}}$ and ${{N_{y} } \mathord{\left/ {\vphantom {{N_{y} } {N_{x} }}} \right. \kern-\nulldelimiterspace} {N_{x} }} = 1$. The decrease of all six lines reveals that the nonlocal effect reduces the critical buckling loads of the nanoplates, and, compared with classical plates, a nanoplate with stronger constraints shows a greater reduction of its critical buckling load. The length effects on the critical buckling load ratios of square SSSS and CCCC nanoplates with different nonlocal parameters are illustrated in Fig. 2b. With the increase of length, it is found that the critical buckling load ratios unanimously increase, and gradually approach 1 that corresponds to the cases of classical plates, which suggests that the nonlocal effect matters for nanoscale plates, but may be negligible for larger-scale plates such that the classical theory can well capture their behaviors.

Defining the frequency ratio as that of the nonlocal theory- to classical theory-based frequencies, Fig. 2c plots the nonlocal parameter dependent fundamental frequency ratios of the six types of square nanoplates with $b = 10\,{\text{nm}}$. The decrease of all six lines reveals that the nonlocal effect reduces the fundamental frequencies of the nanoplates, and, compared with classical plates, a nanoplate with stronger constraints generally shows a greater reduction of its fundamental frequency. The length effects on the fundamental frequency ratios of square SSSS and CCCC nanoplates with different nonlocal parameters are illustrated in Fig. 2d, where the ratios show a dramatical increase, and approach 1 with length, which again suggests that the nonlocal effect plays a significant role for nanoscale plates, but is negligible for macroscale plates.

## Concluding remarks

With an up-to-date symplectic superposition method, the analytic bending, buckling, and free vibration solutions of rectangular nanoplates with combinations of clamped and simply supported edges are obtained based on Kirchhoff plate theory and Eringen’s nonlocal theory. Compared with conventional analytic methods such as the semi-inverse methods, the present method describes the problems in the Hamiltonian system, and yields the analytic solutions by the mathematical techniques in the symplectic space in a rigorous step-by-step way, without predetermining solution forms, which enables one to seek new analytic solutions. After validation of the present method by the other methods, comprehensive benchmark results are presented for both Lévy-type and non-Lévy-type nanoplates, including the bending deflections, critical buckling loads, and natural frequencies. Quantitative parameter analyses are implemented with the analytic solutions to gain insight into the behaviors and to provide reference for structural designs of nanoplates. It should be noted that the present work studies linear free vibration since the adopted superposition technique is applicable in linear regime with small deformation, but the studies on nonlinear vibration are definitely worthy of further exploration, which may constitute our follow-up work.
